# Supplementary material for: Dark state population determines magnetic sensitivity in radical pair magnetoreception model
Source: Sci Rep. 2016 Mar 1;6:22417. doi: 10.1038/srep22417 (PMC4772487; doi:10.1038/srep22417)
Supplement: Supplementary Information [file srep22417-s1.pdf]

Supplementary Information

Dark state population determines magnetic sensitivity in radical pair magnetoreception model

Bao-Ming Xu
School of Physics, Qufu Normal University, Qufu 273165, China

Jian Zou*
School of Physics, Beijing Institute of Technology, Beijing 100081, China

* zoujian@bit.edu.cn

Appendix A: Multi-nuclei RP model

In the main text, the single-nucleus RP model has been discussed, and now we consider the multi-nuclei RP model. Firstly, we only consider the vertical hyperfine interaction, i.e., $A_{nx} = A_{ny} = 0$ for all n. For simplicity, we assume that the hyperfine interaction strengths for all the nuclei are the same, i.e., $A_{nz} = T_z/2$ for all n. In this case the Hamiltonian (see Eq. (1)) in the main text can be expressed as $\hat{H}_0 = \gamma \mathbf{B}\cdot(\hat{S}_1+\hat{S}_2)+T_z\hat{S}_{1z}\hat{J}_z$ with $\hat{J}_z = \sum_n \hat{I}_{nz}/2$. And the initial nuclear state (the completely mixed state) can be expressed as $\rho_I(0) = \frac{1}{2^N}\sum_J\sum_M \nu(N,J)|J,M\rangle\langle J,M|$ where $\nu(N,J) = \binom{N}{N/2-J} - \binom{N}{N/2-J-1}$ denotes the degeneracy of the spin bath with $\binom{N}{-1} = 0$.[1] $|J,M\rangle$ is the eigenvector of $\hat{J}_z$ with $\hat{J}_z|J,M\rangle = M|J,M\rangle$, where $J = 0,1,2,\cdots,N/2$ for N being even, $J = 1/2, 3/2, \cdots, N/2$ for N being odd (N is the total number of the nuclei), $M = -J, -J+1, \cdots, J-1, J$.[2] In this case the effective field induced by the nuclear spins is $MT_z\hat{\mathbf{z}}/\gamma$. The dark state coherence and population can be calculated as

$$\begin{aligned} f_p(t) &= \frac{1}{2^N}\sum_J\sum_M \nu(N,J) f_M^p(t), \\ f_c(t) &= \frac{1}{2^N}\sum_J\sum_M \nu(N,J) f_M^c(t) \end{aligned} \tag{A1}$$

with

$$f_M^p(t) = \frac{1}{2} - \frac{1}{4}\sin^2(\theta_M - \theta)\big[1-\cos(2\omega_M t)\big] \tag{A2}$$

and

$$f_M^c(t) = \frac{1}{2}\cos^4\frac{\theta_M-\theta}{2}\cos\big[2(\omega_M-\omega_0)t\big] + \frac{1}{2}\sin^4\frac{\theta_M-\theta}{2}\cos\big[2(\omega_M+\omega_0)t\big] + \frac{1}{4}\sin^2(\theta_M-\theta)\cos(2\omega_0 t), \tag{A3}$$

where $\omega_M = \gamma B_M$, $B_M = \sqrt{B_x^2 + (B_z + MT_z/\gamma)^2}$, $\sin\theta_M = B_x/B_M$, $\cos\theta_M = (B_z + MT_z/\gamma)/B_M$.

For odd nuclear number N, ignoring the hight frequency oscillating terms, $f_p(t)$ and $f_c(t)$ can be expressed as

$$f_p(t) \approx \frac{1}{2^{N+1}}\sum_J\sum_M \nu(N,J)\left[1 - \frac{1}{2}\sin^2(\theta_M-\theta)\right] \tag{A4}$$

and

$$f_c(t) \approx 0. \tag{A5}$$

Substituting Eq. (A4) (Eq. (A5)) into Eq. (11) (Eq. (12)) in the main text, we obtain

$$\begin{aligned} &\Phi_c \approx 0, \\ &\Phi_p \approx \Phi_s \approx \frac{1}{2^{N+1}}\sum_J\sum_M \nu(N,J)\left[1-\frac{1}{2}\sin^2(\theta_M-\theta)\right]. \end{aligned} \tag{A6}$$

Φ_c is always zero and Φ_p is always equal to Φ_s, which means that the singlet yield is determined completely by the dark state population.

For even nuclear number N, M can equal to 0, so that the hyperfine interaction has no effect on the electrons, i.e., the effective field $MT_z\hat{\mathbf{z}}/\gamma$ induced by the nuclear spins is zero. In this case, $\omega_{M=0} = \omega_0$ and $\theta_{M=0} = \theta$. And there exists a constant term 1/2 in $f_M^c(t)$ (see Eq. (A3)). Neglecting the hight frequency oscillating terms, $f_p(t)$ and $f_c(t)$ can be expressed as

$$f_p(t) \approx \frac{1}{2} - \frac{1}{2^{N+2}}\sum_J\sum_M \nu(N,J)\sin^2(\theta_M-\theta) \tag{A7}$$

and

$$f_c(t) \approx \frac{1}{2^{N+1}}\frac{N!}{((N/2)!)^2}. \tag{A8}$$

Substituting Eq. (A7) (Eq. (A8)) into Eq. (11) (Eq. (12)) in the main text, we obtain

$$\begin{aligned}\Phi_c &\approx \frac{1}{2^{N+1}}\frac{N!}{((N/2)!)^2},\\ \Phi_p &\approx \frac{1}{2}-\frac{1}{2^{N+2}}\sum_J\sum_M \nu(N,J)\sin^2(\theta_M-\theta).\end{aligned} \tag{A9}$$

Φ_c is always a constant and is independent of the geomagnetic field. As a result, although the dark state coherence has contribution to the singlet yield, it does not contribute to the magnetic sensitivity ($\partial\Phi_c/\partial\theta = 0$). This can be understood as follows. When $M = 0$, two electrons are only influenced by the geomagnetic field, and there is no hyperfine interaction to induce the transition between the singlet and triplet states. Therefore for the initial singlet state, its population is unchanged, and thus Φ_c is a constant ($\frac{1}{2^{N+1}}\frac{N!}{((N/2)!)^2}$). Although Φ_p is not equal to Φ_s, $\partial\Phi_p^0/\partial\theta = \partial\Phi_s/\partial\theta$, in another word the magnetic sensitivity is completely determined by $f_p(t)$. It is noted that Φ_c is decreasing with the increasing N, and for a sufficiently large N the singlet yield which comes from the dark state coherence will disappear.

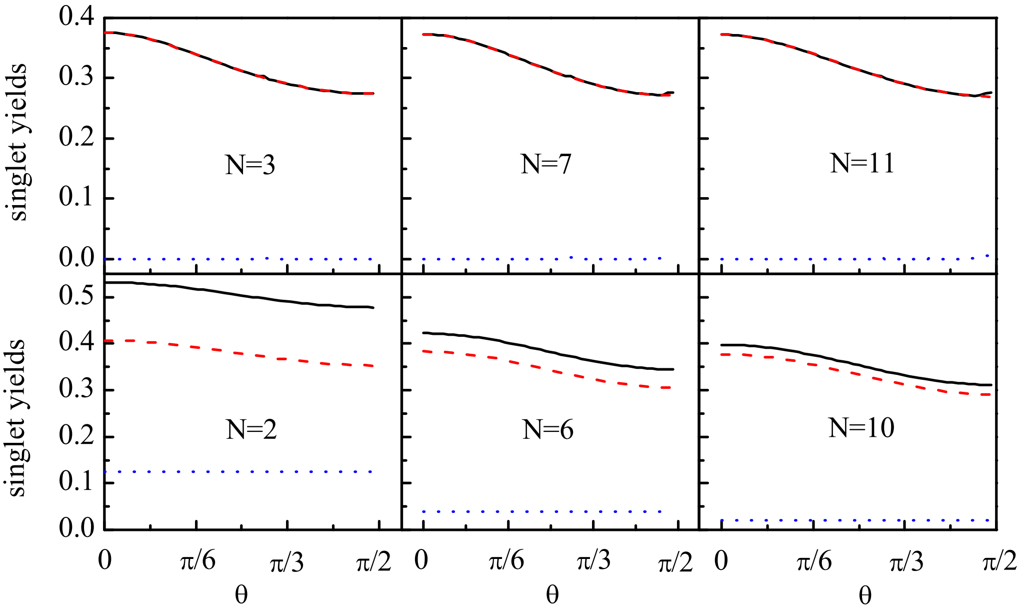

Figure. S1 (Color online) The singlet yields Φ_s (black-solid line), Φ_c (blue-dotted line) and Φ_p (red-dashed line) as functions of the direction angle θ for different N. $T_z = 5\Lambda$, $T_x = 3\Lambda$. It should be noted that Φ_p for the odd nuclear numbers coincides with Φ_s.

Now we consider the horizontal hyperfine interaction, and we still assume that the hyperfine interaction strengths for all the nuclei are the same, i.e., $A_{nx} = A_{ny} = T_x/2$ and $A_{nz} = T_z/2$ for all n. In this case the Hamiltonian (1) in the main text can be expressed as $\hat{H}_0 = \gamma\mathbf{B}\cdot(\hat{S}_1+\hat{S}_2) + T_x(\hat{S}_{1+}\hat{J}_- + \hat{S}_{1-}\hat{J}_+) + T_z\hat{S}_{1z}\hat{J}_z$, where $\hat{S}_{1\pm} = \hat{S}_{1x} \pm i\hat{S}_{1y}$ and $\hat{J}_\pm = \sum_n \hat{I}_{nx} \pm i\hat{I}_{ny}$. It is convenient that we write the initial nuclear spin state (the completely mixed state) in the angular-momentum representation, i.e., $\rho_I(0) = \frac{1}{2^N}\sum_J\sum_M \nu(N,J)|J,M\rangle\langle J,M|$. The action of $\hat{J}_\pm$ on $|J,M\rangle$ is given by $J_\pm|J,M\rangle = \sqrt{(J\pm M+1)(J\mp M)}|J,M\pm 1\rangle$. Setting $T_x = 3\Lambda$ and $T_z = 5\Lambda$ as an example, we numerically calculate the singlet yield as shown in Fig. S1. For N being odd, Φ_c is always 0 for different N and Φ_p always coincides with Φ_s, which means that the singlet yield is determined completely by the dark state population. But if the total number N is even, Φ_c is always a constant (not zero for less N), and Φ_p always differs by a constant from that of Φ_s for all θ, i.e., the magnetic sensitivity $\partial\Phi_p/\partial\theta$ for dark state population is the same as $\partial\Phi_s/\partial\theta$. The distance between Φ_p and Φ_s decreases with the increasing of N. If N is sufficiently large, Φ_c becomes 0 and Φ_p coincides with Φ_s. That is to say, the magnetic sensitivity for even nuclear number is also determined completely by the dark state population.

Appendix B: Two important experimental results

There are two important experimental results which strongly support the RP model. One is that the birds are able to "train" to different field strengths: If the field intensity changes in a suitable regime, for example the field

intensity is increased or decreased by about 30% of the local geomagnetic field, the birds will disorient temporarily but rework after a sufficiently long time to adapt themselves [3–6]. The other is that a very weak oscillating field (generally 150nT or even 15nT) whose frequency is resonant with the electron spin Larmor frequency in the geomagnetic field can disorient the birds completely. But if the oscillatory frequency is detuning from the Larmor frequency, the birds can not be disoriented [7–10]. Now we investigate the influences of the field intensities and the weak oscillating field on the singlet yield from the point of view of the dark state population one by one. For simplicity, we only consider the single-nucleus RP model.

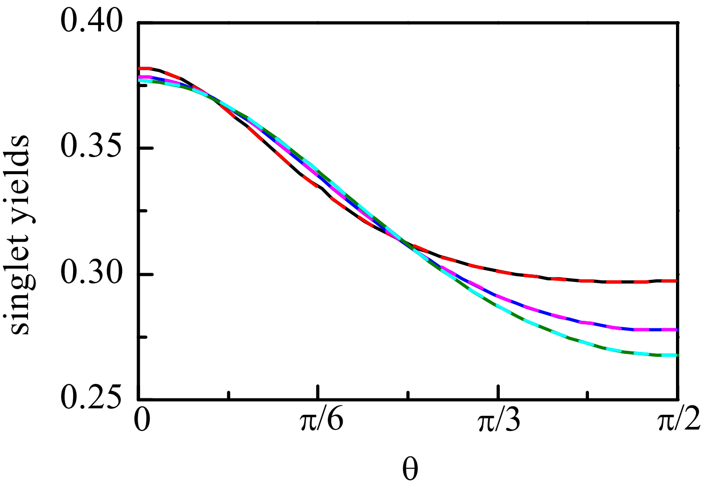

Figure. S2 (Color online) The singlet yields Φ_s (solid lines), Φ_p (dashed lines) as functions of the direction angle θ for different field intensities $B_0 = 32.2\mu$T (olive-solid and cyan-dashed lines), $B_0 = 46\mu$T (blue-solid and magenta-dashed lines), $B_0 = 59.8\mu$T (black-solid and red dashed lines). $A_x = A_y = 3\Lambda$, and $A_z = 5\Lambda$. It should be noted that Φ_p coincides with Φ_s for different field intensities.

Firstly we investigate the effect of the field intensity. For relatively strong hyperfine coupling, we can expand $\sin\theta_\pm$ and $\cos\theta_\pm$ by $\gamma B_0/A_z$, and $f_p(t)$ (see Eq. (17) in the main text) can be expressed as

$$f_p(t) \approx \frac{1}{2} - \frac{1}{4}\sin^2\theta - \frac{\gamma^2 B_0^2}{A_z^2}\left(\frac{3}{4}\sin^2\theta - \sin^4\theta\right). \tag{B1}$$

Substituting Eq. (B1) into Eq. (11) in the main text, one can obtain

$$\Phi_p \approx \frac{1}{2} - \frac{1}{4}\sin^2\theta - \frac{\gamma^2 B_0^2}{A_z^2}\left(\frac{3}{4}\sin^2\theta - \sin^4\theta\right). \tag{B2}$$

It can be seen that the field intensity controls $f_p(t)$ (see Eq. (B1)), thus affects the singlet yield (see Eq. (B2)). The change of the field intensity, for example increasing or decreasing by about 30% of the local geomagnetic field, will induce the change of the singlet yield, so that it disorients the bird transiently. But from Eq. (B2) it can be seen that the singlet yield is mainly determined by $1/2 - \sin^2\theta/4$ which decreases monotonously with θ. And only the second order of $\gamma B_0/A_z$ influences Φ_p (or $f_p(t)$). Although changing the field intensity will change the singlet yield, the monotonicity will be preserved. This monotonicity preservation ensures that the bird reworks after a sufficiently long time to adapt itself. When we consider the horizontal hyperfine interaction $A_x = A_y = 3\Lambda$ and $A_z = 5\Lambda$, we numerically calculate the singlet yield for different magnetic fields as shown in Fig. S2. From Fig. S2 we can see that for different fields Φ_p always coincides with Φ_s along different directions, which means that the dark state population determines the singlet yield completely. We also find that Φ_p (Φ_s) for different field intensities decreases monotonously with the direction angle and the 30% weaker (32.2μT) and stronger (59.8μT) fields influence the angular profile evidently. The changes of the angular profile means that the birds will disorient if the field intensity changes suddenly, but the preservation of the monotonicity ensures that the bird can reorient after a long time to adapt itself.

Next we investigate the influence of the weak oscillating field:

$$\mathbf{B_{rf}} = B_{rf}\cos\omega t(\sin\alpha\cos\beta, \sin\alpha\sin\beta, \cos\alpha), \tag{B3}$$

where $B_{rf} = 150$nT is the strength of the additional oscillating field with frequency ω, and α and β give the direction of the oscillating field. Due to the axial symmetry of the hyperfine interaction tensor we set $\beta = 0$, and only focus on

$\alpha = \theta + \pi/2$, i.e., the radio frequency field is orthogonal to the geomagnetic field. When we only consider the vertical hyperfine coupling, using the time-dependent perturbation theory [11], we can obtain:

$$f_p^{rf}(t) \approx f_p(t) - \frac{1}{16}\gamma^2 B_{rf}^2 \cos^2(\theta_+ - \theta)t^2 - \frac{1}{16}\gamma^2 B_{rf}^2 \cos^2(\theta_- - \theta)t^2. \tag{B4}$$

Substituting Eq. (B4) into Eq. (11) in the main text we can obtain

$$\Phi_p^{rf} \approx \Phi_p - \frac{\gamma^2 B_{rf}^2}{4k^2}\left[\cos^2(\theta_+ - \theta) + \cos^2(\theta_- - \theta)\right]. \tag{B5}$$

It is shown that the weak oscillating field influences the dark state population $f_p(t)$ (see Eq. (B4)), so that destroys the singlet yield anisotropy, and disorients the bird completely. When we consider the horizontal hyperfine coupling $A_x = A_y = 3\Lambda$ and $A_z = 5\Lambda$, we numerically calculate the singlet yield under the influence of the oscillating field as shown in Fig. S3. It can be seen that under the influence of the oscillating field, Φ_p coincides with Φ_s and flattens out. Φ_p coincides with Φ_s means that the singlet yield is still completely determined by the dark state population, and the angular profile flattens out means that under the influence of the resonant radio frequency field the bird can not distinguish different directions θ and disorients completely.

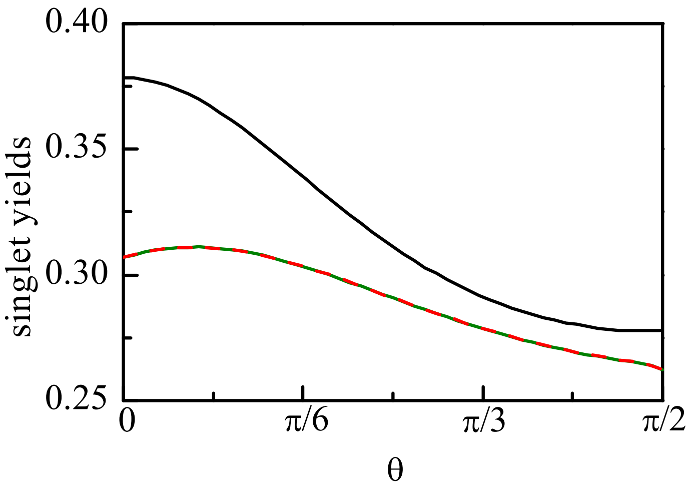

Figure. S3 (Color online) The singlet yields Φ_s^{rf} (olive-solid line) and Φ_p^{rf} (red-dashed line) under the influence of the oscillating field compared Φ_s (black-solid line) without considering the radio frequency field. $B_{rf} = 150$nT, $\omega = 1.315$MHz, $B_0 = 46\mu$T, $A_x = A_y = 3\Lambda$, and $A_z = 5\Lambda$. It should be noted that under the influence of the oscillating field Φ_p^{rf} coincides with Φ_s^{rf}.

Appendix C: Singlet yield anisotropy comes from the initial state anisotropy

Recently, Hogben et al. has pointed out that the singlet yield anisotropy (the singlet yield is different for different θ), is essential to the magnetic sensitivity, which not only can come from the anisotropic hyperfine interaction but also can come from the anisotropic initial state [12]. More specifically, if the initial state is isotropic (for example the singlet state $|S\rangle$), the anisotropic hyperfine interaction ($A_{nx} = A_{ny} \neq A_{nz}$) can induce the singlet yield anisotropy; and if the hyperfine interaction is isotropic ($A_{nx} = A_{ny} = A_{nz}$), the anisotropic initial state (for example the triplet state $|T_0\rangle = (|10\rangle + |01\rangle)/\sqrt{2}$) can also induce the yield anisotropy. The singlet yield anisotropy induced by the anisotropic hyperfine interaction has been investigated in the main text and in Appendix A. For the case that the singlet yield anisotropy is induced by the anisotropic initial state, whether the singlet yield is still completely determined by the dark state population needs to be discussed.

Let two electrons be initially in the triplet state $|T_0\rangle = (|10\rangle + |01\rangle)/\sqrt{2}$ and the hyperfine tensors for single-nucleus and multi-nuclei RPs are $A_x = A_y = A_z = 5\Lambda$ and $A_{nx} = A_{ny} = A_{nz} = 5\Lambda$ for all n, respectively. We numerically calculate the singlet yields for different nuclear number and show the results in Fig. S2. As shown in Fig. S2 for the single-nucleus RP, Φ_p always coincides with Φ_s and Φ_c is always zero for different θ. For the multi-nuclei RP model, if the nuclear number is odd, Φ_p is also coincident with Φ_s and Φ_c is always zero. If the nuclear number is even, there is a little difference between Φ_p and Φ_s. Through our numerical calculation we find that Φ_p is approaching Φ_s and Φ_c

is close to zero as N is increasing. In one word the magnetic sensitivity for the anisotropic initial state and isotropic hyperfine interaction is also determined completely by the dark state population. We also consider other initial states and other hyperfine tensors, and find that the magnetic sensitivity is always determined by the dark state population.

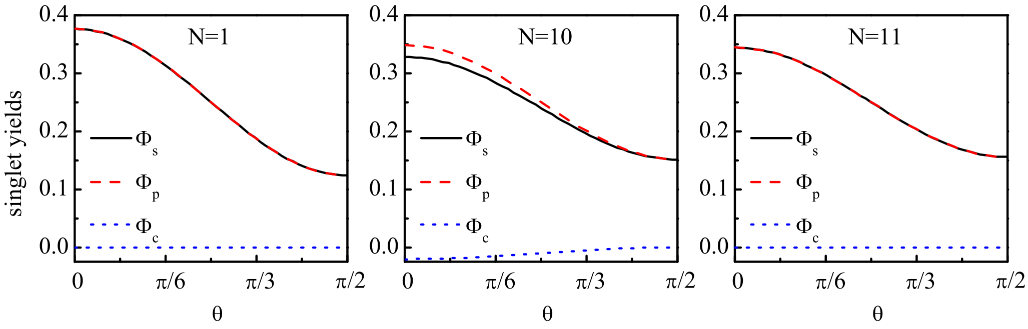

Figure. S4 (Color online) The singlet yields as functions of the direction angle θ for the single-nucleus RP with $A_x = A_y = A_z = 5\Lambda$ and the multi-nuclei RP model with $A_{nx} = A_{ny} = A_{nz} = 5\Lambda$ for all n. Two electrons are initially in the triplet state $|T_0\rangle = (|10\rangle + |01\rangle)/\sqrt{2}$. It should be noted that Φ_p for odd nuclear numbers coincide with Φ_s.

Appendix D: Derivation of the master equation

Considering the classical noise, the total Hamiltonian is

$$H(t) = H_0 + H^{'}(t) = H_0 + h(t)\hat{h}. \tag{D1}$$

In the interaction picture, the Liouville's equation can be written as ($\hbar = 1$)

$$\frac{d}{dt}\rho_I(t) = -i[H_I(t),\ \rho_I(t)], \tag{D2}$$

where $\rho_I(t) = e^{iH_0 t}\rho(t)e^{-iH_0 t}$, $H_I(t) = e^{iH_0 t}H^{'}(t)e^{-iH_0 t} = h(t)e^{iH_0 t}\hat{h}e^{-iH_0 t} = h(t)\hat{h}_I(t)$. Generally, Eq. (D2) can be solved by iteration,[13, 14]

$$\rho_I(t) = \rho_I(0) - i\int_0^t dt_1 h(t_1)[\hat{h}_I(t_1), \rho_I(0)] - \int_0^t dt_1 \int_0^{t_1} dt_2 h(t_1)h(t_2)[\hat{h}_I(t_1), [\hat{h}_I(t_2), \rho_I(0)]] + \cdots. \tag{D3}$$

Due to the noise, the ensemble average density matrix satisfies the following equation:

$$\bar{\rho}_I(t) = \rho_I(0) - i\int_0^t dt_1 \langle h(t_1)\rangle[\hat{h}_I(t_1), \rho_I(0)] - \int_0^t dt_1 \int_0^{t1} dt_2 \langle h(t_1)h(t_2)\rangle[\hat{h}_I(t_1), [\hat{h}_I(t_1), \rho_I(0)]] + \cdots. \tag{D4}$$

We consider a Gaussian white noise, i.e., $\langle h(t)\rangle = 0$, thus the n'th-order correlation can be written as

$$\langle h(t_1)h(t_2)\cdots h(t_n)\rangle = \begin{cases} 0 & \text{if } n \text{ is odd,} \\ \sum\limits_{\substack{all\ \ (n-1)!! \\ pairings}} \langle h(t_1)h(t_2)\rangle\langle h(t_3)B^{'}(t_4)\rangle \cdots \langle h(t_{n-1})h(t_n)\rangle & \text{if } n \text{ is even,} \end{cases} \tag{D5}$$

with $(n-1)!! = (n-1)(n-3)\cdots 5\cdot 3\cdot 1$.[15] We assume that $\langle h(t)h(\tau)\rangle = \Gamma\delta(t-\tau)$, i.e., the Markovian process, and obtain

$$\bar{\rho}_I(t) = \rho_I(0) - \int_0^t dt_1 \Gamma[\hat{h}_I(t_1), [\hat{h}_I(t_1),\ \rho_I(0)]] + \int_0^t dt_1 \Gamma^2[\hat{h}_I(t_1), [\hat{h}_I(t_1), \int_0^{t_1} dt_2[\hat{h}_I(t_2), [\hat{h}_I(t_2), \rho_I(0)]]]] + \cdots, \tag{D6}$$

which is just the iterative expression of the following differential equation,[13, 14]

$$\frac{d}{dt}\bar{\rho}_I(t) = -\Gamma[\hat{h}_I(t), [\hat{h}_I(t), \bar{\rho}_I(t)]]. \tag{D7}$$

In the Schrödinger picture, it can be written as

$$\frac{d}{dt}\bar{\rho}(t) = -i[H_0, \bar{\rho}(t)] - \Gamma[\hat{h}, [\hat{h}, \bar{\rho}(t)]]. \tag{D8}$$

[1] Schliemann, J., Khaetskii, A. & Loss, D. Electron spin dynamics in quantum dots and related nanostructures due to hyperfine interaction with nuclei. *J. Phys.: Condens. Matter* **15**, R1809 (2003).

[2] Gross, M. & Haroche, S. Superradiance: an essay on the theory of collective spontaneous emission. *Phys. Rep.* **93**, 301 (1982).

[3] Wiltschko, W. & Wiltschko, R. Magnetic compass of European robins. *Science* **176**, 62 (1972).

[4] Wiltschko, W. *in Animal Migration, Navigation, and Homing, edited by Schmidt-Koenig K. & Keeton, W. T.* (Springer, New York, 1978).

[5] Wiltschko, W., Stapput, K., Thalau, P. & Wiltschko, R. Avian magnetic compass: fast adjustment to intensities outside the normal functional window. *Naturwissenschaften* **93**, 300 (2006).

[6] Winklhofer, M., Dylda, E., Thalau, P., Wiltschko, W. & Wiltschko, R. Avian magnetic compass can be tuned to anomalously low magnetic intensities. *Proc. R. Soc. B* **280**, 20130853 (2013).

[7] Ritz, T., Thalau, P., Phillips, J. B., Wiltschko, R. & Wiltschko, W. Resonance effects indicate a radical-pair mechanism for avian magnetic compass. *Nature* **429**, 177 (2004).

[8] Thalau, P., Ritz, T., Stapput, K., Wiltschko, R. & Wiltschko, W. Magnetic compass orientation of migratory birds in the presence of a 1.315 MHz oscillating field. *Naturwissenschaften* **92**, 86 (2005).

[9] Ritz, T. *et al.* Magnetic Compass of Birds Is Based on a Molecule with Optimal Directional Sensitivity. *Biophys. J.* **96**, 3451 (2009).

[10] Wiltschko, R. *et al.* Magnetoreception in birds: the effect of radio-frequency fields. *J. R. Soc. Interface* **12**, 20141103 (2015).

[11] Xu, B. M., Zou, J, Li, H., Li, J. G. & Shao, B. Effect of radio frequency fields on the radical pair magnetoreception model. *Phys. Rev. E* **90**, 042711 (2014).

[12] Hogben, H. J., Biskup, T. & Hore, P. J. Entanglement and Sources of Magnetic Anisotropy in Radical Pair-Based Avian Magnetoreceptors. *Phys. Rev. Lett.* **109**, 220501 (2012).

[13] Loreti, F. N. & Balantekin, A. B. Neutrino oscillations in noisy media. *Phys. Rev. D* **50**, 4762 (1994).

[14] Wang, Z. S. Geometric Phase in Fluctuating Magnetic Field. *Int. J. Theor. Phys.* **48**, 2353 (2009).

[15] Mandel, L. & Wolf, E. *Optical coherence and quantum optics* (Cambridge University Press, Cambridge, 2011).
